# Attitudes and use of complementary and alternative medicine: a cross-sectional comparison between medical and non-medical students

**DOI:** 10.3389/fphar.2025.1529079

**Published:** 2025-09-12

**Authors:** Maryam Zakeri, Mahlagha Dehghan, Yaser Soltanmoradi, Shiva Monfared, Gulsah Kose, Hojjat Farahmandnia, Alaa Hamza Hermis, Xiao Xu, Mohammad Ali Zakeri

**Affiliations:** ^1^ Pistachio Safety Research Center, Rafsanjan University of Medical Sciences, Rafsanjan, Iran; ^2^ Reproductive and Family Health Research Center, Kerman University of Medical Sciences, Kerman, Iran; ^3^ Department of Operating Room Technology, Faculty member, School of Paramedicine, Rafsanjan University Medical of Sciences, Rafsanjan, Iran; ^4^ Social Determinants of Health Research Center, Rafsanjan University of Medical Sciences, Rafsanjan, Iran; ^5^ Mugla Sıtkı Kocman University, Faculty of Health Sciences, Department of Nursing, Mugla, Türkiye; ^6^ Health in Disasters and Emergencies Research Center, Institute for Futures Studies in Health, Kerman University of Medical Sciences, Kerman, Iran; ^7^ College of Nursing, Al-Qadisiyah University, AL-Dewaynia, Iraq; ^8^ Department of Nursing, Nantong Health College of Jiangsu Province, Nantong, China; ^9^ Molecular Medicine Research Center, Research Institute of Basic Medical Sciences, Rafsanjan University of Medical Sciences, Rafsanjan, Iran; ^10^ Clinical Research Development Unit, Ali-Ibn Abi-Talib Hospital, Rafsanjan University of Medical Sciences, Rafsanjan, Iran

**Keywords:** attitudes, opinions, complementary, alternative, medicine

## Abstract

Students are one of the groups in society that use complementary and alternative medicine (CAM). Given their role in promoting the use of CAM and the potential differences in attitudes due to their educational backgrounds, it is important to investigate their perspectives on CAM. This study aimed to compare the attitudes and use of CAM between medical and non-medical students. The study employed a cross-sectional design and was conducted among 525 medical and non-medical students in Iran. Data were collected using a descriptive information form, a CAM questionnaire, and the Holistic Complementary and Alternative Medicine Questionnaire (HCAMQ). The data were analyzed using SPSS version 25. The mean HCAMQ scores for medical and non-medical students were 33.10 ± 5.39 and 31.96 ± 5.48, respectively. A significant difference was found between medical and non-medical students in terms of their attitudes toward CAM, as measured by the HCAMQ subscale (p = 0.005). Additionally, 72.8% of medical students and 61.0% of non-medical students reported using at least one CAM method. A positive and statistically significant relationship was observed between medical and non-medical students in terms of their overall use of CAM (p = 0.005) and nutritional supplement methods (p = 0.025). Analysis of the reasons for using CAM revealed that only the use of medicinal herbs showed a significant difference between medical and non-medical students (p = 0.002). Finally, there was no statistically significant difference between the two groups in terms of consulting a physician before using CAM methods. Given the significant difference in attitudes toward CAM between medical and non-medical students, it is essential to address the distinct educational needs of these two groups. Developing effective and targeted educational programs could improve their knowledge and promote the safe and informed use of CAM.

## 1 Introduction

Complementary and alternative medicine (CAM) refers to a group of systems, practices, and products that are not typically considered part of conventional medical methods (Babar, Syed, Naing and Hamzah, 2012; Pham, Yoo, Tran and Ta, 2013; Samara, Barabra, Quzaih and Zyoud, 2019). CAM encompasses treatments that are either complementary to or substitutes for conventional drug therapies (Jafari, Zanganeh, Kazemi, Lael-Monfared and Tehrani, 2021). Literally, the term “alternative” refers to practices that replace conventional medical treatments, while “complementary” refers to those that are used alongside conventional treatments (Samara et al., 2019). The National Center for CAM classifies CAM therapies into categories such as “alternative medicine systems, mind-body interventions, biologically based therapies, body-based and manipulative methods, and energy therapies” (Singh and Kamath, 2021). In some cultures, certain CAM methods, including prayer and Quran recitation, which fall outside these categories, are widely popular among the general population (Dehghan, Ghanbari, Heidari, Mangalian and Zakeri, 2021; Samara et al., 2019). The classification of prayer and Quran recitation as CAM aligns with World Health Organization (WHO) benchmarks that encompass traditional therapeutic practices. The WHO Traditional Medicine Strategy underscores the cultural and historical significance of such modalities and emphasizes the integration of Traditional and Complementary Medicine (TCM) into national health systems. This strategy aims to provide a framework for the safe and effective delivery of TCM across diverse health contexts (Permatasari, Hasina and Pratama, 2020; Zeid, Andersen and Kristiansen, 2019).

According to the WHO, 75% of the global population still relies on traditional medical systems (Singh and Kamath, 2021). The prevalence of CAM usage ranges from 9.8% to 76% (Ashraf et al., 2019), and its popularity continues to grow (Singh and Kamath, 2021; Steel et al., 2018). This increasing interest in CAM is attributed to its perceived effectiveness, safety, cost-effectiveness, and alignment with personal, religious, and spiritual beliefs (Ashraf et al., 2019; Iktidar et al., 2022). CAM is often self-administered or initiated under the supervision of a specialist (Steel et al., 2018).

Studies on CAM have identified several factors driving its use, including the rising cost of modern medical care, dissatisfaction with conventional treatments, a desire for greater personal involvement in health management, holistic health beliefs, and improved access to CAM services (El-Olemy, Radwan, Shihab and Dawood, 2014). The primary users of CAM include patients with conditions such as asthma, arthritis, psoriasis, cancer, chronic liver disease, irritable bowel syndrome, and gastroesophageal reflux (Yurtseven et al., 2015; Zeidabadi, Abbas, Mangolian Shahrbabaki and Dehghan, 2022). Commonly used CAM methods worldwide include herbal medicine, spiritual healing/prayer, massage therapy, acupuncture, hypnosis, meditation, vitamins, and homeopathy (Ashraf et al., 2019).

In Iran, CAM is widely used, with approximately 95% of cancer patients and 31% of dermatology patients relying on it (Jafari et al., 2021). During the stressful COVID-19 pandemic, herbal and complementary medicine used to prevent and treat illness, as well as to strengthening the immune system against the virus (Nguyen, De Tran, Pham, Dao and Dewey, 2021). In support of this finding, numerous studies have shown that CAM can be effective in reducing symptoms, treatment and prevention COVID-19 infection (Kretchy et al., 2021; Nilashi, Samad, Yusuf and Akbari, 2020; Wu, Dong, Chi, Yu and Wang, 2021). However, evidence for the effectiveness of most CAM interventions still needs evaluation (Jeon et al., 2022).

COVID-19, known as acute respiratory syndrome, is a viral disease characterized by symptoms such as fever, fatigue, muscle pain, diarrhea, and, in severe cases, lung damage (Bulatova et al., 2022). Social distancing remains the primary method for preventing COVID-19 infection (Badakhsh et al., 2021), while treatment for infected individuals includes rest and adequate fluid intake (Dehghan et al., 2021). Despite the success of vaccines and drug therapies in combating COVID-19, interest in CAM remains high, and a wide range of CAM methods have been proposed and investigated as preventive and therapeutic measures against the virus (Bulatova et al., 2022). Given the increasing prevalence of COVID-19, burnout among healthcare workers, and the lack of definitive treatments, drugs, equipment, and manpower particularly in less developed countries CAM approaches with minimal side effects may serve as preventive measures, especially for mild cases, which constitute the majority of patients (Badakhsh et al., 2021).

Public awareness and the use of CAM are complex phenomena that have grown significantly over the past decade. One reason for this dramatic increase is the widespread exposure to information through the internet and mass media. Commercial advertisements, official press, medical journals, and books have promoted the use of complementary medicine, particularly among younger generations who show a keen interest in CAM (El-Olemy et al., 2014). Various studies have reported positive attitudes toward CAM among medical students (Ameade, Amalba, Helegbe and Mohammed, 2016; Rotter, Jerzynski, Hinse, Binting and Brinkhaus, 2021). Similarly, studies on Iranian medical and pharmacy students have found that they possess sufficient knowledge of and exhibit positive attitudes toward CAM (Parvinroo, Daeihamed, Rafiei and Tajik, 2021; Sadeghi, Rabiepoor, Forough, Jabbari and Shahabi, 2016). As part of the younger generation in every society, medical students may play a significant role in promoting the use of CAM. However, access to vast amounts of information from diverse sources, coupled with the lack of formal education about CAM, may lead to its incorrect use among students (El-Olemy et al., 2014; Iktidar et al., 2022). Therefore, it is crucial to investigate the attitudes and usage of CAM among both medical and non-medical students.

This study can help identify educational gaps among medical and non-medical students and improve educational programs. By examining students’ attitudes toward CAM, there may be a need for further education on scientific evidence or the development of educational programs that provide accurate, evidence-based information about CAM. This can reduce misuse and increase awareness. A better understanding of attitudes and the use of CAM can lead to the effective integration of these practices into modern medicine, benefiting both patients and healthcare systems. Medical students represent future healthcare providers whose CAM attitudes may influence clinical practice; non-medical students reflect public perspectives that shape demand. This comparison highlights gaps in CAM education and public health outreach.

Additionally, this study can contribute to the development of ethnopharmacology, offering a deeper understanding of the interaction between culture, traditional medicine, and modern medicine. However, only a limited number of studies dealt with the attitudes of non-medical students towards CAM. Besides, no studies compared the attitudes of medical and non-medical students towards CAM and their use of CAM. Within this context, this study aims to compare the opinions, attitudes and use of CAM between the medical and non-medical students.

## 2 Materials and methods

### 2.1 Study design and participants

This study had a cross-sectional design to compare the attitudes and use of CAM between the medical and non-medical students. Convenience sampling method was used to access potential participants. Cochran’s formula calculated a minimum sample size of 384 participants for Confidence factor = 95 percent; p = q = 0.5; Z = 1/96 (d = 0.05). Voluntary university students above the age of 18 years, who lived in Iran, had access to social networks and completed all questions of the online questionnaire sent by the researchers were included to the study. Medical students were defined as those enrolled in medical schools pursuing a medical degree, while non-medical students were those enrolled in other disciplines (e.g., engineering, humanities). Participants were recruited through social media platforms (WhatsApp, Telegram and other national social networks) and university groups. Questionnaires were administered in Persian, and Google Forms were used for data collection. The study was concluded with the participation of 525 students.

### 2.2 Data collection tools

Descriptive information form, CAM questionnaire, and HCAMQ were used for data collection. The CAM questionnaire assesses general attitudes and usage patterns of CAM, while the HCAMQ provides a more nuanced understanding of holistic health beliefs (Supplementary Material).

#### 2.2.1 Descriptive information form

The form was prepared by the researchers and included questions on age, gender, marital status, education level, chronic diseases, addiction and COVID-19 infection and vaccination.

#### 2.2.2 Complementary and alternative medicine (CAM) questionnaire

The 10 item CAM questionnaire was developed by Dehghan et al. to investigate the use of CAM methods in Persian language. This questionnaire consists of three parts: a) Amount of use CAM (herbal medicines, nutritional supplements, wet cupping, dry cupping, massage, acupuncture, acupressure, homeopathy, relaxation techniques such as yoga and prayer): Items were scored on a 7-item Likert scale (0 = from never/rarely, 6 = every day). b) Reason for use CAM: Reasons to use CAM were also measured using three options: reducing physical symptoms, reducing anxiety and stress, and others. c) Consultation with physician/medical staff for use CAM: A yes/no question asked if the participant consulted a doctor before using CAM. Cronbach’s alpha of the questionnaire was 0.85 (Dehghan et al., 2020). The 0–6 frequency scale was selected to differentiate non-users (0) from minimal users (≥1), enhancing clinical interpretability. The 6-point agreement scale omitted a neutral midpoint to reduce ambivalence, following forced-choice methodology. In the present study, participants reported using prayer and supplication as part of CAM practices. In Islamic culture, prayer and supplication are integral to spiritual and therapeutic approaches aimed at enhancing physical and mental wellbeing. In Islam, “Dua” refers to the act of seeking help from God to alleviate problems, illnesses, and stress. This can include reciting specific verses from the Quran, particular invocations, or daily prayers as regular spiritual practices to reduce stress and promote psychological tranquility. However, our study did not specify the particular type of prayer or supplication used. These practices are often employed as complementary to modern medical treatments to improve overall health. Within the framework of the present study, the term “dietary supplements” refers to vitamins, minerals, probiotics, or commercially available formulations consumed orally to enhance dietary intake. Medicinal herbs are defined as plant-derived products utilized for therapeutic purposes, including teas, extracts, or raw botanical materials (e.g., chamomile, turmeric).

#### 2.2.3 Holistic Complementary and Alternative Medicine Questionnaire (HCAMQ)

The 11-item HCAMQ was developed by Hyland et al. to measure beliefs about the scientific validity of CAM (6 items) and holistic health (HH) about CAM (5 items) in English language (Hyland, Lewith and Westoby, 2003). Items were scored on a 6-point Likert scale ranging from strongly agree (1) to strongly disagree (6). Possible scores ranged from 11 to 66, with lower scores reflected a more pro-attitude toward HH and CAM. The test-retest reliability score of the total HCAMQ was 0.86 (Hyland et al., 2003). After obtaining permission, HCAMQ were translated into Persian using forward-backward translation, with discrepancies resolved by a panel of bilingual experts. Ten faculty members of the Rafsanjan Faculty of Nursing and Midwifery were asked to review the questionnaire for content validity and we found that the content validity index of the questionnaire was 0.90 and the Cronbach’s alpha coefficient was 0.87.

### 2.3 Data collection

An online survey, which included the descriptive information form, CAM questionnaire, and HCAMQ was sent to the participants via social networks between February to May 2021. After providing informed consent, the participants could answer each question for once. The online survey was completed by 525 students. The use of online convenience sampling may favor students with greater CAM awareness or access to digital platforms. While this limits generalizability, it reflects pragmatic constraints during data collection.

### 2.4 Data analysis

SPSS version 24 software was used for data analysis. Descriptive data were presented in frequency and percentage. Independent t-test and analysis of variance were used to determine the relationship between descriptive information and the scores obtained from the CAM questionnaire. Independent t-test was used to compare the HCAMQ scores obtained by the medical and non-medical students whereas Fisher’s Exact Test and Chi-Square test were used to compare the scores obtained from the CAM questionnaire. Statistical significance was set at 0.05.

### 2.5 Ethical issue

We obtained approval from the Ethics Committee of Rafsanjan University of Medical Sciences (IR.RUMS.REC.1401.096). Informed consent was obtained online from all participants before completing the online survey. Participants were informed about the scope of the study and confidentiality was secured. All participant data were anonymized during collection and analysis. Electronic responses were stored on password-protected servers compliant.

## 3 Results

### 3.1 Characteristics of participants

The samples included 294 medical and 231 non-medical students with a mean age of 21.84 ± 3.17 and 23.97 ± 6.11 years, respectively. In both groups of students, females outnumbered males, but the proportion of females was higher among medical students (n = 205; 69.7%). The majority of participants were single, although the proportion of married individuals was greater among non-medical students (n = 48; 21.3%). In both groups of students, the majority were at the undergraduate level. The prevalence of chronic diseases and addiction was low in both groups, and there was no significant difference between the two groups (Table 1).

**TABLE 1:** Demographic characteristics (n = 525).

Variable	Frequency (%)	Medical students (n = 294)	Non-medical students (n = 231)
		Frequency (%)	Frequency (%)
Gender			
Male	180 (34.3)	89 (30.3)	91 (39.4)
Female	345 (65.7)	205 (69.7)	140 (60.6)
Marital status^a^			
Married	85 (16.5)	37 (12.8)	48 (21.3)
Single	430 (83.5)	253 (87.2)	177 (78.7)
Education level			
Associate degree	50 (9.5)	15 (5.1)	35 (15.2)
Bachelor’s degree	394 (75.0)	239 (81.3)	155 (67.1)
Masters and Ph.D	81 (15.4)	40 (13.6)	41 (17.7)
Chronic disease^a^			
Yes	17 (3.3)	8 (2.8)	9 (4.0)
No	496 (96.7)	281 (97.2)	215 (96.0)
Addiction^a^			
Yes	11 (2.1)	6 (2.1)	5 (2.3)
No	502 (97.9)	285 (97.9)	217 (97.7)
Infected with COVID-19			
Yes	234 (44.6)	128 (43.5)	106 (45.9)
No	291 (55.4)	166 (56.5)	125 (54.1)
COVID-19 vaccination			
Yes	507 (96.6)	289 (98.3)	218 (94.4)
No	18 (3.4)	5 (1.7)	13 (5.6)

^a^ Missing value.

Social networks and internet were used by the majority of students medical (n = 206; 70.1%) and non-medical students (n = 150; 64.9%) to obtain information about CAM. Regarding the sources of information about CAM, there was a significant difference between the medical and non-medical students, who utilized health professionals for information (p = 0.001) (Table 2).

**TABLE 2:** Sources of information about CAM (n = 525).

Variable		Frequency (%)	Medical students (n = 294)	Non-medical students (n = 231)	Test Statistic	p value
Radio and TV	Yes	208 (39.6)	118 (40.1)	90 (39.0)	*χ* ^*2*^ = 0.07	0.78
	No	317 (60.4)	176 (59.9)	141 (61.0)		
Book	Yes	37 (7.0)	18 (6.1)	19 (8.2)	*χ* ^*2*^ = 0.87	0.39
	No	488 (93.0)	276 (93.9)	212 (91.8)		
Social networks and internet	Yes	356 (67.8)	206 (70.1)	150 (64.9)	*χ* ^*2*^ = 1.56	0.22
	No	169 (32.2)	88 (29.9)	81 (35.1)		
Health professionals	Yes	226 (43.0)	146 (49.7)	80 (34.6)	*χ* ^*2*^ = 11.91	0.001*
	No	299 (57.0)	148 (50.3)	151 (65.4)		
Friends	Yes	230 (43.8)	125 (42.5)	105 (45.5)	*χ* ^*2*^ = 0.45	0.53
	No	295 (56.2)	169 (57.5)	126 (54.5)		
Satellite	Yes	23 (4.4)	14 (4.8)	9 (3.9)	*χ* ^*2*^ = 0.23**	0.67
	No	502 (95.6)	280 (95.2)	222 (96.1)		
Others	Yes	65 (12.4)	32 (10.9)	33 (14.3)	*χ* ^*2*^ = 1.38	0.28
	No	460 (87.6)	262 (89.1)	198 (85.7)		

TV: television, χ2: Chi-Square test, **: Fisher’s Exact Test.

Bold value indicates the significance level, *p < 0.05.

### 3.2 Comparing use of CAM between medical and non-medical students

Table 3 showed that 72.8% (n = 214) of the medical students used at least one method of CAM, including medicinal herbs (n = 132; 44.9%), nutritional supplements (n = 109; 37.1%) and prayer (n = 89; 30.3%). On the other hand, 61.0% (n = 141) of the non-medical students used at least one method of CAM of CAM, including medicinal herbs (n = 96; 41.6%), nutritional supplements (n = 64; 27.7%) and prayer (n = 61; 26.4%). There was a positive and statistically significant relationship between total CAMs (p = 0.005) and nutritional supplement usage (p = 0.025) of the medical and non-medical students.

**TABLE 3:** Methods of CAM used by medical and non-medical students (n = 525).

Variable	Medical students (n = 294)		Non-medical students (n = 231)		Test Statistic	p value
	Frequency (%)	Confidence interval (%)	Frequency (%)	Confidence interval (%)		
Medicinal herbs	132 (44.9)	39.8–50.7	96 (41.6)	35.5 _ 48.0	0.58^a^	0.47
Dry cupping	5 (1.7)	0.3–3.1	7 (3.0)	0.9 _ 5.6	1.02^a^	0.38
Wet cupping	6 (2.0)	0.7–3.7	6 (2.6)	0.9 _ 5.2	0.17^a^	0.77
Massage	17 (5.8)	3.4–8.5	10 (4.3)	2.2 _ 6.9	0.56^a^	0.55
Nutritional supplements	109 (37.1)	31.3–42.9	64 (27.7)	21.6 _ 33.3	5.14^a^	0.025*
Acupuncture	3 (1.0)	0.0–2.4	3 (1.3)	0.0 _ 3.0	0.08^b^	1.00
Acupressure	3 (1.0)	0.0–2.4	4 (1.7)	0.4 _ 3.5	0.49^b^	0.70
Hemeopathy	4 (1.4)	0.3–2.7	4 (1.4)	0.4 _ 3.5	0.11^b^	0.73
Relaxation and meditation	35 (11.9)	8.2–15.6	18 (7.8)	4.3 _ 11.7	2.41^a^	0.14
Prayer	89 (30.3)	25.2–35.7	61 (26.4)	21.2 _ 32.0	0.94^a^	0.38
Total CAM	214 (72.8)	67.7–78.2	141 (61.0)	54.5 _ 67.5	8.15^a^	0.005*

^a^ Chi-Square test, ^b^Fisher’s Exact Test.

Bold value indicates the significance level, *p < 0.05.

We found that 61.5% (n = 64) and 47.7% (n = 61) of the medical students used nutritional supplements and medicinal herbs for the prevention of COVID-19 disease, respectively. Besides, 40.2% (n = 35), 18.8% (n = 24) and 54.3% (n = 19) of these students used prayer, medicinal herbs and relaxation & meditation to reduce stress and anxiety, respectively. On the other hand, 56.4% (n = 75) and 55.9% (n = 33) of the non-medical students used nutritional supplements and medicinal herbs for prevention of COVID-19 disease, respectively. Additionally, 45.8% (n = 27) of these students have used prayer to reduce stress and anxiety, respectively.

Nutritional supplements were used by the medical students (n = 64) to prevent COVID-19 disease whereas medicinal herbs were utilized by the non-medical students with the same purpose (n = 75). On the other hand, prayer was the most used method of CAM by the medical (n = 35) and non-medical (n = 27) students to reduce stress and anxiety.

The analysis of the reasons to utilize CAMs, we found that only the use of medicinal herbs had a significant difference between medical and non-medical students (p = 0.002) (Table 4).

**TABLE 4:** Reasons for using different methods of CAM (n = 525).

Variable	Medical students (n = 294)			Non-medical students (n = 231)			Test Statistic	p value
	Prevention of covid-19 disease	Reducing stress and anxiety	Others	Prevention of covid-19 disease	Reducing stress and anxiety	Others		
Medicinal herbs	61 (47.7)	24 (18.8)	43 (33.6)	75 (56.4)	6 (4.5)	52 (39.1)	13.00^a^	0.002*
Dry cupping	3 (60.0)	1 (20.0)	1 (20.0)	2 (28.6)	0 (0.0)	5 (71.4)	3.63^a^	0.16
Wet cupping	3 (50.0)	1 (16.7)	2 (33.3)	1 (16.7)	1 (16.7)	4 (66.6)	1.66^a^	0.43
Massage	2 (11.8)	5 (29.4)	10 (58.8)	0 (0.0)	4 (44.4)	5 (55.6)	1.45^a^	0.48
Nutritional supplements	64 (61.5)	7 (6.7)	33 (31.7)	33 (55.9)	2 (3.4)	24 (40.7)	1.82^a^	0.40
Acupuncture	1 (33.3)	1 (33.3)	1 (33.4)	1 (33.3)	0 (0.0)	2 (66.7)	0.66^b^	0.50
Acupressture	0 (0.0)	2 (66.7)	1 (33.3)	0 (0.0)	2 (50.0)	2 (50.0)	0.19^b^	0.62
Hemeopathy	1 (25.0)	2 (50.0)	1 (25.0)	0 (0.0)	0 (0.0)	3 (100.0)	3.93^a^	0.14
Relaxation and meditation	3 (8.6)	19 (54.3)	13 (37.1)	1 (5.6)	5 (27.8)	12 (66.7)	4.18^a^	0.12
Prayer	14 (16.1)	35 (40.2)	38 (43.7)	6 (10.1)	27 (45.8)	26 (44.1)	1.15^a^	0.56

^a^ Chi-Square test, ^b^Fisher’s Exact Test, Others: strengthening the immune system, decreasing fatigue. Bold value indicates the significance level, *p < 0.05.

### 3.3 Comparing consultation with a physician for using CAM between medical and non-medical students

We found that 70.5% (n = 74) of the medical students consulted with a physician to use medicinal herbs whereas 28.9% (n = 37) consulted for nutritional supplements. The percentages for medicinal herbs and nutritional supplements in non-medical students were 37.3% (n = 50) and 59.0% (n = 36), respectively. There was no statistically significant relationship between the method of CAM and consultation with a physician in both the medical and non-medical students (Table 5).

**TABLE 5:** Consultation with a physician for using method of CAM (n = 525).

Variable	Medical students (n = 294)		Non-medical students (n = 231)		Test Statistic	p value
	Yes	No	Yes	No		
Medicinal herbs	37 (28.9)	91 (71.1)	50 (37.3)	84 (62.7)	2.08^a^	0.15
Dry cupping	1 (20.0)	4 (80.0)	3 (42.9)	4 (57.1)	0.68^b^	0.57
Wet cupping	3 (60.0)	2 (40.0)	4 (66.7)	2 (33.3)	0.05^b^	>0.99
Massage	6 (35.3)	11 (64.7)	5 (55.6)	4 (44.4)	0.99^b^	0.41
Nutritional supplements	74 (70.5)	31 (29.5)	36 (59.0)	25 (41.0)	2.26^a^	0.17
Acupuncture	1 (33.3)	2 (66.7)	2 (66.7)	1 (33.3)	1.33^a^	0.51
Acupressture	2 (66.7)	1 (33.3)	2 (66.7)	1 (33.3)	0.00^a^	>0.99
Hemeopathy	2 (50.0)	2 (50.0)	1 (33.3)	2 (66.7)	0.19^b^	>0.99
Relaxation and meditation	9 (28.1)	23 (71.9)	2 (11.8)	15 (88.2)	1.70^a^	0.28
Prayer	8 (9.3)	78 (90.7)	5 (8.8)	52 (91.2)	0.01^a^	>0.99

^a^ Chi-Square test, ^b^Fisher’s Exact Test.

### 3.4 Comparing attitudes of CAM between medical and non-medical students

The mean HCAMQ scores obtained by the medical and non-medical students were 33.10 ± 5.39 and 31.96 ± 5.48, respectively. The mean HCAMQ score of the medical students was statistically significantly higher than non-medical students (p = 0.017). Besides, there was a statistically significant difference between the scores obtained from the attitudes towards CAM subscale (p = 0.005). On the other hand, there was no significant difference between the scores obtained by the two groups from the holistic health subscale of the HCAMQ (Table 6).

**TABLE 6:** Comparison of the HCAMQ scores (n = 525).

Group Variables	Medical students (n = 294)		Non-medical students (n = 231)		Independent t-test	p value
	Mean	Standard deviation	Mean	Standard deviation		
HCAM	33.10	5.39	31.96	5.48	2.38	0.017*
Attitudes towards CAM	19.00	3.87	18.04	3.85	2.80	0.005*
Holistic health	14.10	2.75	13.91	2.85	0.76	0.446

HCAM: Holistic Complementary and Alternative Medicine.

Bold value indicates the significance level, *p < 0.05.

## 4 Discussion

The present study was conducted to investigate and compare the attitudes and the use of CAM in medical and non-medical students.

The results showed that medical students had higher attitude scores and a more positive view of CAM compared to non-medical students. Similarly, Ashraf et al. found that pharmacy students held more positive beliefs and attitudes toward complementary medicine than non-pharmacy students (Ashraf et al., 2019). Additionally, Akan et al. in Turkey reported that medical students exhibited positive attitudes toward the use of CAM (Akan et al., 2012). Al Mansour et al. in Saudi Arabia also documented a positive attitude toward CAM among medical students (Al Mansour et al., 2015).

Contrary to our finding, Iktidar et al. reported that non-medical students had a more favorable attitude towards complementary medicine than medical students (Iktidar et al., 2022). Similarly, Alshahrani’s study in Saudi Arabia found no significant differences in attitudes toward CAM between medical and non-medical students (Alshahrani, 2024). Medical students may have more favorable attitudes and greater knowledge of CAM due to their educational exposure. Ashraf et al.'s study showed that, despite the lack of formal education on CAM and limited knowledge, pharmacy students demonstrated better practical knowledge of herbal medicine compared to non-pharmacy students (Ashraf et al., 2019). This may be attributed to their greater educational exposure. Furthermore, some studies have indicated that students believe CAM should be made more scientific. Numerous reports in the literature suggest that medical students support the inclusion of CAM in their curricula to guide and improve its practice in professional healthcare delivery (Hussain et al., 2012; James, Bah and Kondorvoh, 2016). Although there is a need to revise academic curricula for medical students to incorporate CAM education, it is equally important to inform and educate non-medical students. Improving their attitudes and knowledge about CAM can ensure its correct and effective use.

We found a significant difference between medical and non-medical students in terms of the general use of CAM and nutritional supplements. Consistent with our findings, Liu et al. reported that medical students had a more positive attitude and a higher rate of using nutritional supplements compared to non-medical students (Liu et al., 2018). However, no other studies on this issue have been identified, highlighting the need for further research to confirm our findings. Given the influence of Iranian culture, which is often conveyed through social and entertainment media, students may have a greater familiarity with practices such as yoga, which originates from India, a neighboring country. This underscores the importance of cultural context in shaping knowledge and attitudes toward CAM.

In our study, 72.8% of medical students and 61.0% of non-medical students reported using at least one method of CAM, including medicinal plants, nutritional supplements, and prayer. Similarly, Alshahrani’s study in Saudi Arabia found that herbal medicine was the most commonly used CAM method among medical students (21%), while spiritual healing/Quran recitation was the most prevalent among non-medical students (19%) (Alshahrani, 2024). Ashraf et al. identified medicinal herbs, massage, spiritual healing, yoga, and homeopathy as the most common CAM methods among pharmacy and non-pharmacy students (Ashraf et al., 2019). Additionally, Iktidar et al. reported that homeopathy was the most widely known CAM method among medical students (44.6%), while herbal medicine was the most popular among non-medical students (45.7%) (Iktidar et al., 2022). Other studies have also highlighted medicinal plants and food supplements as the most frequently used CAM methods among medical students and healthcare personnel (Ameade et al., 2016; Bulatova et al., 2022; Samara et al., 2019). However, the preferred CAM methods vary across countries. For example, magnetic therapy and spiritual healing are the most well-known CAM methods in Saudi Arabia, medicinal plants are the most popular in Turkey (Bishop, Yardley and Lewith, 2006) and herbal medicines were more popular in Kuwait (Ilori, Akintayo, Adewale and Oyetola, 2021). These differences can be attributed to variations in cultural and belief systems (Iktidar et al., 2022; Yurtseven et al., 2015).

In the present study, the internet and social networks were the primary sources of information about CAM for both medical and non-medical students. Similarly, Yurtseven et al. found that the internet (52.2%) and television (27.2%) were the main sources of CAM information (Yurtseven et al., 2015). Iktidar et al. reported that medical and non-medical students in Bangladesh relied on friends, personal experience, and newspapers as their primary sources of CAM information (Iktidar et al., 2022). Ashraf et al. identified family, friends, and mass media as the most common sources of CAM information among pharmacy and non-pharmacy students (Ashraf et al., 2019). Given the widespread accessibility of the internet and the vast amount of content available online (Jafari et al., 2021), it is reasonable to expect the internet to be a primary source of information. However, we found a significant difference between medical and non-medical students in terms of utilizing healthcare professionals as a source of information. To date, no other studies have identified healthcare professionals as a significant source of CAM information.

Regarding the reasons for using CAM, there was a significant difference between medical and non-medical students in terms of using medicinal plants. Ilori et al. found that the majority of participants in Nigeria used CAM to mitigate the adverse psychological effects of COVID-19 (Ilori et al., 2021). Kichen et al. reported that nursing students used prayer, meditation, and traditional medicine to manage stress caused by their work and study environments (Kinchen and Loerzel, 2019). EL-Olemy et al. found that spiritual healing and Quran recitation were commonly used by Egyptian non-medical students and their families for direct healing purposes (El-Olemy et al., 2014). Since the reasons for using CAM vary across cultures, it is essential to pay closer attention to the attitudes of individuals in different communities to ensure the correct and effective use of CAM.

There was no significant difference between medical and non-medical students in terms of consulting a physician before using CAM methods. Tozun et al.'s study on Turkish healthcare personnel and medical students found that consultation was one of the three main sources of information about CAM methods (Tozun, Bag, Pektas, Soyacikgoz and Tekindal, 2022). In contrast, Liu et al. reported that the majority of medical and non-medical students used supplements without consulting a physician (Kinchen and Loerzel, 2019). However, the number of studies investigating CAM use among non-medical students remains limited, underscoring the need for further research to validate our findings. Additionally, given the importance of consulting a physician before using CAM methods, it is crucial to educate CAM users about potential side effects through social media and other accessible platforms (Zakeri, Bagheripour, Iriti and Dehghan, 2021; Zakeri, Mohammadi, Bazmandegan and Zakeri, 2020).

## 5 Limitations

This study has several limitations. Conducted in a single country, its findings may lack generalizability to university students in other cultural contexts. As data were collected online, participation bias may have favored students with greater pre-existing interest in CAM, potentially inflating positive attitudes. Future studies should employ stratified random sampling across universities to enhance representativeness. Methodologically, Questionnaire 1 utilized a 7-point Likert scale (0–6) with “0”(never/rarely) capturing absolute non-use. However, the absence of a midpoint risks oversimplifying frequency gradations. Similarly, the 6-point agreement scale may have amplified extreme responses by excluding a neutral option, potentially overestimating CAM-related attitudes. Future surveys should implement balanced scales (e.g., 5- or 7-point) to capture nuanced perspectives, and compare forced-choice versus neutral-inclusive designs. Furthermore, this study did not assess CAM knowledge a critical limitation when interpreting attitudes. Future research should evaluate knowledge levels to determine whether favorable views reflect evidence-based understanding or cultural familiarity. Convenience sampling restricts generalizability beyond digitally engaged populations, while self-reported data may introduce social desirability bias. Finally, the temporal context of the COVID-19 pandemic may have increased CAM usage; thus, further studies are needed to confirm these findings.

## 6 Conclusion

The results of the present study indicate favorable attitudes toward CAM and a high level of CAM use among both medical and non-medical students¶. Therefore, identifying the different educational needs of these two groups and developing appropriate educational programs tailored to these needs could improve students’ knowledge of CAM, particularly among non-medical students. Additionally, training on CAM should emphasize the conscious and safer use of available complementary medicine methods. National guidelines must be advocated to standardize CAM education, and policymakers should ensure alignment with broader healthcare policies. Collaborative efforts among medical institutions, CAM practitioners, and regulatory bodies are imperative to harmonize educational content with clinical practice. Furthermore, programs to familiarize stakeholders with region-specific CAM practices must be prioritized, alongside addressing transnational challenges such as the proliferation of misinformation regarding CAM use. Attention must be directed toward challenges in CAM curriculum development and budgeting, the necessity of CAM instruction by trained faculty, and cultural-regulatory heterogeneity, particularly in regions where CAM is deeply culturally entrenched. Longitudinal studies to assess the impact of CAM education on clinical practice and to investigate cultural and social determinants of attitudes toward CAM are essential in future research.

## Data Availability

The original contributions presented in the study are included in the article/Supplementary Material, further inquiries can be directed to the corresponding author.
