# Supplementary material for: Attitudes and use of complementary and alternative medicine: a cross-sectional comparison between medical and non-medical students
Source: Front Pharmacol. 2025 Sep 12;16:1529079. doi: 10.3389/fphar.2025.1529079 (PMC12464550; doi:10.3389/fphar.2025.1529079)
Supplement: Supplementary file 1 [file Table1.docx]

**به نام خدا**

**دانشجوی گرامی**

**پرسشنامه زیر به‌منظور بررسی نگرش و کاربرد برخی از انواع طب مکمل و جایگزین در دانشجویان ایرانی تنظیم‌شده است ضمن تشکر از شما، خواهشمندم سؤالات زیر را به‌دقت مطالعه و پاسخ دهید.**

**1-سن: .......... 2-جنس: .......... 3-وضعیت تاهل: ..........**

**4- نوع دانشگاه : علوم پزشکی 🔿 غیر علوم پزشکی 🔿**

**5- مقطع تحصیلات: کاردانی 🔿 کارشناسی 🔿 کارشناسی ارشد و بالاتر 🔿**

**6- آیا به بیماری خاصی مبتلا هستید؟ خير 🔿 بلي 🔿**

**7- آیا به ماده خاصی اعتیاد دارید؟ خير 🔿 بلي 🔿**

**8- آیا به بیماری کرونا مبتلا شده اید: خير 🔿 بلي 🔿**

**9- آیا شما واکسن کرونا را تزریق کرده اید: خير 🔿 بلي 🔿**

**10- اطلاعات خود در خصوص استفاده از طب مکمل را چگونه بدست آورده اید: (بیش از یک مورد را می توانید انتخاب کنید)**

**1. شبکه های اجتماعی 🔿 2. سایت بهداشتی و درمانی 🔿 3. پرسنل و کادر بهداشتی و درمانی 🔿 4. کتاب و مجلات 🔿 5. رادیو و تلویزیون 🔿 6. دوستان و آشنایان 🔿 7. اطلاعاتی دریافت نکرده ام 🔿**

**لطفاً در صورتی که از هریک از روش های زیر را استفاده کرده اید، به دقت به موارد ذکر شده در جدول پاسخ دهید.**

| **روش** | **میزان استفاده** | | | | | | | **علت استفاده** | | | **آیا با پزشک/کادر درمان در این خصوص مشورت کرده اید؟** | |
| --- | --- | --- | --- | --- | --- | --- | --- | --- | --- | --- | --- | --- |
|  | **هرگز** | **به ندرت** | **ماهی یک بار** | **2 بار در ماه** | **یک بار در هفته** | **2 تا 3 بار در هفته** | **هر روز** | **پیشگیری از ابتلا به کرونا** | **بهبود اضطراب و استرس** | **سایر موارد (نام ببرید)** | **بلی** | **خیر** |
| **1. داروهای گیاهی (نام داروهای گیاهی مورد استفاده را ذکر کنید)** |  |  |  |  |  |  |  |  |  |  |  |  |
| **2. بادکش** |  |  |  |  |  |  |  |  |  |  |  |  |
| **3. حجامت** |  |  |  |  |  |  |  |  |  |  |  |  |
| **4. ماساژ** |  |  |  |  |  |  |  |  |  |  |  |  |
| **5. مکمل های غذایی (نام مکمل مورد استفاده را ذکر کنید)** |  |  |  |  |  |  |  |  |  |  |  |  |
| **6. طب سوزنی** |  |  |  |  |  |  |  |  |  |  |  |  |
| **7. طب فشاری** |  |  |  |  |  |  |  |  |  |  |  |  |
| **8. هومئوپاتی** |  |  |  |  |  |  |  |  |  |  |  |  |
| **9. شیوه های مختلف آرام سازی مانند یوگا** |  |  |  |  |  |  |  |  |  |  |  |  |
| **10. دعا و توسل و نذر** |  |  |  |  |  |  |  |  |  |  |  |  |

**لطفاٌ در صورتی که از شیوه های دیگری استفاده کرده اید نام ببرید. ...................................**

**سؤالات زیر ممکن است برای شما دشوار باشند، اما آنها تجربیات معمول متخصصان مراقبت های بهداشتی هستند. آنها به تجربیات شما در حرفه شما و اینکه اکنون چه احساسی دارید مربوط می شود. سعی کنید به هر سوالی پاسخ دهید. دور یک عدد بین 1 و 6 (کاملاً موافقم = 1، موافقم = 2، کمی موافقم = 3، کمی مخالفم = 4، مخالفم = 5، کاملاً مخالفم = 6) خط بکشید تا مشخص کنید که شخصاً چقدر با هر عبارت موافق یا مخالف هستید.**

| **پرسشنامه** | **کاملا موافقم** | **موافقم** | **کمی موافقم** | **کمی مخالفم** | **مخالفم** | **کاملا مخالفم** |
| --- | --- | --- | --- | --- | --- | --- |
| **1- تفکر مثبت می تواند به شما کمک کند تا با یک بیماری معمولی مقابله کنید.** |  |  |  |  |  |  |
| **2- داروی مکمل قبل از پذیرش توسط طب معمول باید تحت آزمایشات علمی بیشتری قرار گیرد.** |  |  |  |  |  |  |
| **3- وقتی افراد دچار استرس می شوند، مهم است که آنها در مورد سایر جنبه های زندگی خود مراقب باشند زیرا بدن آنها توانایی کافی برای کنار آمدن با ان را برخوردار است.** |  |  |  |  |  |  |
| **4- داروی مکمل از این نظر خطرناک است که ممکن است از درمان مناسب افراد جلوگیری کند.** |  |  |  |  |  |  |
| **5- علائم یک بیماری می تواند با افسردگی بدتر شود.** |  |  |  |  |  |  |
| **6- داروهای مکمل فقط باید به عنوان آخرین راه حل مورد استفاده قرار بگیرند زمانی که داروهای مرسوم ارائه نمی شوند.** |  |  |  |  |  |  |
| **7- اگر فردی یک سری وقایع استرس زای زندگی را تجربه کند، آنها بیشتر بیمار می شوند.** |  |  |  |  |  |  |
| **8- ارزش دارد قبل از مراجعه به پزشک داروی مکمل را امتحان کنید.** |  |  |  |  |  |  |
| **9- درگیری با دیگران تاثیری در سلامتی شما ندارد.** |  |  |  |  |  |  |
| **10- داروی مکمل فقط باید در بیماری های معمولی استفاده شود و نه در درمان بیماری های جدی تر.** |  |  |  |  |  |  |
| **11- ایجاد تعادل بین کار و استراحت برای حفظ سلامتی اهمیت دارد.** |  |  |  |  |  |  |
| **12- داروی مکمل باعث ایجاد دفاع در بدن می شود، بنابراین منجر به درمان دائمی می شود.** |  |  |  |  |  |  |
